# Modelling human proteostasis and organelle homeostasis disorders in yeast and its application in drug discovery

**DOI:** 10.1093/femsyr/foag023

**Published:** 2026-06-02

**Authors:** Anh V Do, Memoona Zahra, Alan L Munn

**Affiliations:** School of Environment and Science, Griffith University, Gold Coast Campus, Southport, QLD 4222, Australia; Griffith Institute for Biomedicine and Glycomics, Griffith University, Gold Coast Campus, Southport, QLD 4222, Australia; School of Pharmacy and Medical Sciences, Griffith University, Gold Coast Campus, Southport, QLD 4222, Australia; School of Pharmacy and Medical Sciences, Griffith University, Gold Coast Campus, Southport, QLD 4222, Australia

**Keywords:** enzyme replacement therapy, snake venom, yeast two-hybrid, chemical genomics, amyloid, lysosome

## Abstract

Disruptions in cellular homeostasis and proteostasis are central to many human diseases, yet direct mechanistic investigation in human systems remains constrained by biological complexity and ethical limitations. Therefore, researchers have turned to the use of model systems that allow the more efficient dissection of fundamental cellular processes. The unicellular yeast *Saccharomyces cerevisiae* has emerged as a powerful eukaryotic model for studying disorders driven by defects in homeostasis and proteostasis. The relevant processes are highly conserved in yeast, enabling precise genetic manipulation and real-time analysis of mechanisms that are difficult to study in mammalian systems. Yeast models have been deployed to study prion propagation, lysosomal enzyme trafficking, and mitochondrial dysfunction. Yeast also provides a versatile platform for drug discovery, particularly through the use of the yeast two-hybrid system and high-throughput screens. Despite an inability to recapitulate the full complexity of multicellular organisms, yeast remains an invaluable tool for investigating human diseases and for the development of therapeutics. This review highlights how yeast has uniquely advanced the understanding of human diseases including those associated with prions, lysosomal proteins, and mitochondria and can be combined with the utility of yeast in drug discovery—collectively establishing yeast as a model for studying human disorders.

## Teaser

This reviews applications of yeast to both understanding human disease and advancing disease treatment that have not previously been reviewed and cites some specific examples (e.g. snake bite, lysosomal disease enzyme replacement therapy). Created in BioRender. Do, A. (2026) https://BioRender.com/yo2zglb

## Introduction

Defects in proteostasis and organelle function represent central drivers of many human diseases, particularly neurodegenerative disorders and metabolic disorders (Morimoto 2008). Investigating the cellular mechanisms underlying these disorders has been greatly advanced through study of genetically tractable eukaryotic model systems. Among these, *Saccharomyces cerevisiae*, commonly known as baker’s yeast, is a unicellular fungus and contains around 6000 genes, of which 5570 are predicted to be protein-encoding (Goffeau et al. 1996, Wood et al. 2001). These features enable yeast to function as an experimental model for multiple human diseases, including neurodegenerative conditions, metabolic diseases and cancer (Smith and Snyder 2006, Miller-Fleming et al. 2008).

Many pathways governing proteostasis and organelle function are conserved in yeast, e.g. cell death regulation, mitochondrial biology, DNA repair, and protein folding (Lasserre et al. 2015, Bilinski et al. 2017). Its high degree of conservation in these fundamental cellular processes allows yeast to be a valuable asset for biomedical research. As a eukaryote, yeast has a compact genome with less gene redundancy, which simplifies genetic manipulation and supports large-scale genome engineering projects, including construction of systematic gene deletion libraries for chemical genomic studies where the growth of gene knockout strain is examined in the presence of different compounds to reveal drug targets and mechanism of action (Giaever and Nislow 2014, Sharma 2020). *Saccharomyces cerevisiae* possesses features that facilitate systematic analysis of pathological variants and high-throughput chemical genetic screening which further enhances its utility in laboratory settings (Stepchenkova et al. 2023). The yeast genome is highly amenable to modification, allowing researchers to introduce human genes, delete wild-type yeast genes or engineer yeast to express human mutant proteins to investigate the effects of infectious agents (Dunham and Fowler 2013). Additionally, yeast’s ability to alternate between haploid and diploid life cycles is a unique advantage in genetic analysis. Importantly, research involving yeast is not subject to the stringent ethical regulations that govern animal or human studies, enabling more efficient experimentation. These attributes collectively establish yeast as an exceptional model for uncovering the molecular underpinnings of human diseases.

## Yeast as a genetically tractable model for human disease and proteostasis mechanism

The genetic tractability of *S. cerevisiae* makes it an excellent model for investigating human disease-associated genes and their functions. Introducing human genes into yeast allows researchers to study their function under diverse stress conditions, such as heat shock or oxidative stress or starvation (Sharma 2020). This allows researchers to examine the effects and mutations in a controlled environment, thus enabling the understanding of genetic interactions.

In eukaryotes, mitochondria are essential organelles responsible for production of adenosine triphosphate (ATP) through oxidative phosphorylation as well as regulating metabolite synthesis and apoptotic signalling (Saraste 1999). Yeast mitochondria are structurally and functionally similar to human mitochondria, including the organisation of the electron transport chain, mitochondrial DNA maintenance, and core metabolic pathways. This unique feature of *S. cerevisiae* allows the adaptation to severe mitochondrial dysfunction with impaired oxidative phosphorylation while remaining viable. This enables the systematic analysis of pathogenic mutations that would be otherwise lethal in mammalian cells. Through this, yeast provides a more coherent platform for investigating molecular impacts regarding disease-associated cellular dysfunction, including increased production of reactive oxygen species and altered respiratory function (Lasserre et al. 2015).

### Modelling amyloid-associated diseases in yeasts

Amyloids are not a distinct class of protein but rather a three-dimensional structural state characterised by misfolded proteins that aggregate into highly ordered cross-β sheet fibrils. Under certain conditions, including neuroinflammation, genetic mutations, or infections, proteins can transform into an amyloid form. While most amyloids are non-infectious, in some cases, such as prions, the amyloid state itself can become transmissible and propagate between cells (Greenwald and Riek 2010, Toyama and Weissman 2011). Normal protein synthesis is largely driven by ribosomes and their availability and efficiency are essential rate-limiting factors for cell growth and division (Yu and Nielsen 2020). In this cellular process, misfolded proteins can propagate their structure by inducing normal proteins to misfold (McAlary et al. 2019, Casey and Sleator 2024). The key feature of this conformation change is that the predominantly α-helical structure of the functional protein adopts the predominantly β-sheet-rich structure of the aberrant protein (Prusiner 1998). This chain reaction causes more infectious proteins to be generated, resulting in an accumulation of aggregates that is ultimately characterised by the formation of plaques in the brain, causing neuroinflammation and cell death (Ogomori et al. 1989, Wickner 1994, Prusiner 1998).

### The nature of prion diseases

**Figure 1.**
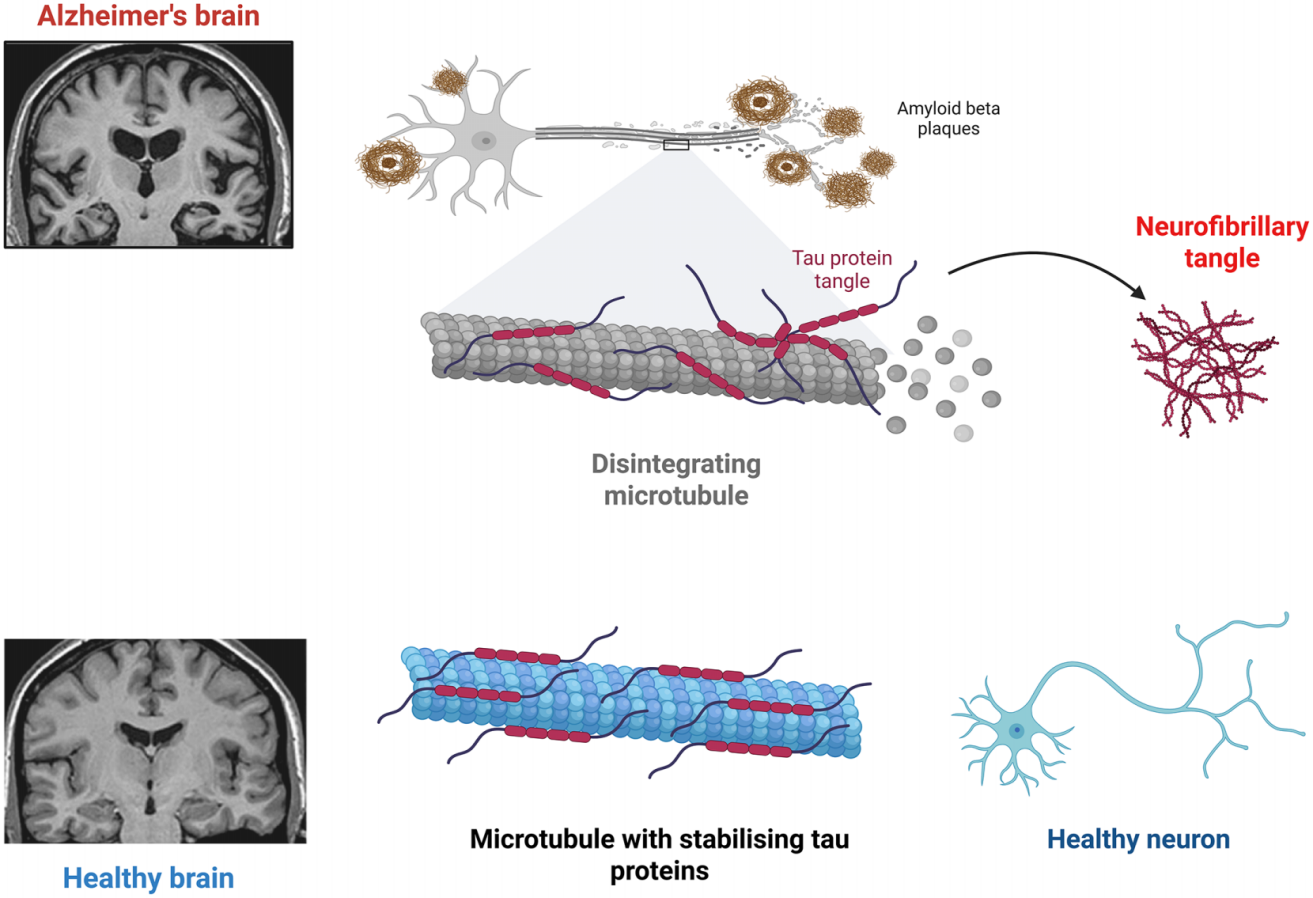
A graphical comparison of a healthy brain and its neurons to a brain affected by Alzheimer’s disease, which contains tau plaques in its neurons. Created in BioRender. Do, A. (2026) https://BioRender.com/ktihsiv

Prions are considered a subclass of amyloid proteins distinguished by their ability to self-propagate and misfold when they become infectious (Wickner et al. 2007). In humans, prions manifest in diseases such as kuru, fatal familial insomnia (FFI), Creutzfeldt–Jakob disease (CJD), the recently described variably protease-sensitive prionopathy (VPSPr), as well as transmissible spongiform encephalopathies (TSEs) in cattle (Prusiner 1998, Atkinson et al. 2016, Casey and Sleator 2024). These diseases arise from the conformational conversion of normal cellular prion protein (PrP^C^), into a disease-associated isoform called scrapie prion protein (PrP^Sc^). The normal form of protein PrP^C^ is an extremely conserved cell surface glycosylphosphatidylinositol (GPI)-anchored sialoglycoprotein having a structure in which the C-terminal domain contains three α-helices, a small anti-parallel β-sheet and the flexible N-terminal domain is disordered (Wüthrich and Riek 2001, Rodriguez et al. 2017). This isoform, found exclusively in prion-infected tissues, forms detergent-insoluble, polymeric aggregates that are often resistant to protease digestion (Prusiner 1987, Prusiner 1991). Unlike typical infectious agents, PrP^Sc^ lacks nucleic acid and propagates through the self-templated conversion of PrP^C^ into an aberrant, aggregation-prone β-sheet-rich structure. This process leads to the formation of insoluble amyloid assemblies that spread within the nervous system and are associated with progressive neuronal dysfunction and loss (Fig. 1) (Riesner 2003, Eghiaian et al. 2004, Soto and Satani 2011).

Structural studies of yeast prions, including [*PSI^+^*], [URE3], and [*PIN^+^*], revealed that these amyloid fibres adopt a folded in-register parallel β-sheet architecture, in which identical residues align along the fibril axis (Wickner et al. 2008, Kraus et al. 2021). This structural organization provides a molecular explanation for conformational templating, as incoming monomers are guided into the same misfolded state through residue-to-residue interactions. Notably, recent high-resolution structural analysis of infectious mammalian prions by Kraus et al. demonstrated that PrP-based prions adopt a similarly ordered β-sheet–rich fibrillar architecture, providing direct structural support for the templating mechanism originally defined in yeast prions and strengthening the mechanistic link between yeast models and mammalian disease (Kraus et al. 2021).

Human prion diseases can originate through three distinct routes: sporadically, as in sporadic Creutzfeldt-Jakob disease, which can occur without any known trigger; via inherited autosomal dominant mutations in the *PRNP* gene that predispose the protein to misfold; and acquired through exposure to infectious prions, either through dietary sources, such as bovine spongiform encephalopathy (BSE)-contaminated beef or through iatrogenic pathways, including infected organs or tissues being used for transplants or contaminated medical and surgical instruments (Collinge 2001). Regardless of the route of origin, these diseases share hallmark neuropathological features, including characteristic spongiform changes such as synaptic loss, axonal degeneration, and neuronal cell death, often accompanied by gliosis and amyloid plaque deposition. A defining aspect of prion disorders is their prolonged incubation period, during which pathogenic changes silently progress before clinical symptoms emerge. As the disease advances, the degeneration of neurons triggers the release of inflammatory mediators—such as cytokines, reactive oxygen species, and proteases—that exacerbate neuronal injury and contributes to the loss of damaged cells.

### Prions in yeast and their relevance to human disease

In 1965, Brian Cox discovered a non-chromosomal genetic element in *S. cerevisiae* called [*PSI⁺*], which enhanced the efficiency of translation readthrough at termination codons (Cox 1965). Several years later, François Lacroute described another non-Mendelian determinant, [URE3], which mimicked the phenotype of a *ure2* loss-of-function mutant, allowing yeast cells to utilise ureidosuccinate as a nitrogen source despite the presence of preferred nitrogen sources (Lacroute 1971).

In 1994, Reed Wickner proposed that the non-Mendelian genetic element [URE3] in budding yeast *S. cerevisiae* is a prion form of the Ure2 protein. His genetic evidence showed that [URE3] propagation depends on the presence of Ure2p and can be cured by guanidine hydrochloride treatment. Wickner hypothesised that [URE3] represents an altered, self-propagating conformation of Ure2p that converts the normal protein into the same inactive form, establishing the first protein-based inheritance model in a micro-organism (Wickner 1994). In the same study, Wickner further proposed that the [*PSI⁺*] determinant represents a prion form of Sup35p, based on genetic evidence paralleling that for [URE3] and building on earlier experiments by Cox, Ter-Avanesyan, and Chernoff (Cox 1965, Wickner 1994, Ter-Avanesyan et al. 1994, Paushkin et al. 1996, Derkatch et al. 1996, Dergalev et al. 2019).

Although yeast does not possess a direct homolog of PrP^C^, several yeast proteins, including Ure2p and Sup35p, have been identified that can adopt either a normal soluble state or a transmissible amyloid conformation (Liebman and Chernoff 2012, Wickner et al. 2015, Wickner et al. 2018). In [*PSI⁺*] and [URE3] strains, the native proteins Sup35p and Ure2p aggregate in a self-propagating manner that can be triggered via inherited cytoplasmically during cell division, where the misfolded protein state is transmitted from mother to daughter cells through the partitioning of prion aggregates. Notably, the prion domain or the full-length protein can become aberrant in vitro, which facilitates the transmission of the prion [URE3] and [*PSI^+^*] upon introduction into non-aberrant cells (Tanaka et al. 2004, Liebman and Chernoff 2012). For a protein to be classified as a prion, it needs to have an essential region called a prion domain (PrD). The main characteristic of a prion domain is that it consists of peptide sequences that are rich in two amino acids: glutamine (Q) and asparagine (N). For this reason, the prion domain regions are also known as Q/N-rich domains (Alberti et al. 2009). Previous research established that prion domains are crucial to a prion protein as they play a role in prion-forming ability, and this is particularly useful for identifying new prions. (Wickner 2019). However, not every Q/N-rich domain is capable of generating a prion, indicating that additional sequence context and structural features are required. For instance, the yeast prion protein Ure2p contains an N-terminal prion-forming domain enriched in glutamine and asparagine residues that is sufficient to drive [URE3] prion formation independently of the remainder of the protein. In contrast, the C-terminal domain retains its role in nitrogen catabolite regulation when overexpressed. (Wickner et al. 2007, 2015)

In addition to revealing mechanisms of prion formation and propagation, studies in yeast have uncovered multiple cellular anti-prion systems that limit the emergence and persistence of prions. These pathways involve conserved proteostasis factors that promote curing or suppress toxicity, ensuring that the vast majority of newly arising prions are eliminated before they compromise cellular viability. Several components of these anti-prion networks have identifiable homologues in higher eukaryotes, suggesting that similar protective mechanisms may operate in human cells. Moreover, recent work has demonstrated that expression of specific human proteins in yeast can suppress or eliminate yeast prions, further highlighting the translational relevance of yeast as a model for understanding cellular defence strategies against protein misfolding (Wickner 2019).

The presence of prion domains explains the intrinsic ability of Sup35p and Ure2p to adopt self-propagating prion conformations, however, for the prion states to achieve maintenance and inheritance, it requires an active cellular machinery. In yeast, most prions propagation critically depend on the molecular chaperone Hsp104 (apart from [*GAR^+^*] and [*ISP^+^*]), a member of the heat-inducible Clp/Hsp100 family of AAA + ATPases first identified in *Escherichia coli*. Hsp104 contains two nucleotide-binding domains (NBD1 and NBD2), whose ATPase activity drives the fragmentation of prion aggregates, a process essential for their stable inheritance (Parselt et al. 1991, Chernoff et al. 1995, Moriyama et al. 2000). Unlike conventional chaperones that primarily prevent aggregation, Hsp104 remodels protein assemblies by disassembling stress-induced aggregates and restoring functional proteins when cellular proteostasis systems become saturated (Bösl et al. 2006). In the context of prions, Hsp104 acts by fragmenting large amyloid assemblies into smaller, seeding-competent units, thereby generating multiple transmissible prion particles that can be efficiently partitioned into daughter cells during cell division (Satpute-Krishnan et al. 2007). Stable propagation of [*PSI⁺*] requires tightly regulated Hsp104 activity, as both loss of function and overexpression lead to prion curing. Hsp104 is similarly required for the maintenance of other yeast prions. This role extends to other yeast prions, including [URE3] and the prion-like element [*PIN⁺*], highlighting a conserved function of Hsp104 in prion propagation and inheritance (Wegrzyn et al. 2001). Controlled amyloid fragmentation by Hsp104 is therefore essential for balancing prion transmission and stability (Shorter and Lindquist 2008). The balance between aggregate fragmentation and complete disassembly therefore, determines whether a prion state is stably propagated or eliminated (Pezza and Serio 2007). These observations demonstrated that prion inheritance is not a passive consequence of protein aggregation, but rather a regulated process controlled by cellular protein quality-control systems, establishing yeast as a uniquely powerful model for dissecting the mechanistic basis of prion biology.

### Exploring human amyloidoses through the lens of yeast prions

Alzheimer’s disease, Parkinson’s disease, Huntington’s disease, and serum amyloid A (AA) amyloidosis are among several human conditions that display prion-like characteristics such as seeding, misfolding and propagating observed in cellular models, animal systems and human pathological tissues (Cascarina and Ross 2014). Although there is no evidence that these disorders are contagious through ordinary social contact, credible evidence indicates that amyloid pathology can be transmitted in both clinical and laboratory settings under rare iatrogenic circumstances (Kim et al. 2019, Banerjee et al. 2024, Singh et al. 2024). In particular, between 1959 and 1985, there were at least 1 848 patients who underwent treatment with human cadaveric pituitary-derived growth hormone (c-hGH) during childhood in the United Kingdom (Swerdlow et al. 2003). As surveillance expanded, these incidents were later associated with the development of over 200 cases of iatrogenic CJD documented worldwide and ∼80 cases occurred in the United Kingdom. Evidence for iatrogenic transmission of amyloid pathology in humans has emerged from several clinical observations. Follow-up analyses of historical cadaveric human growth hormone (c-hGH) preparations revealed the presence of tau and amyloid-β (Aβ), which retained the ability to seed amyloid pathology in mouse models. Similarly, cases of iatrogenic Creutzfeldt–Jakob disease (CJD) following dural mater grafting have been associated with an unusually high prevalence of Aβ deposition in meningeal vessels of relatively young patients lacking a family history of early-onset dementia or significant tau pathology, raising the possibility of a causal link to prior dural grafting (Frontzek et al. 2016).

One explanation for these observations is that Aβ aggregates introduced via medical procedures undergo prolonged incubation and subsequent propagation within host tissues, resembling the seeding and spread mechanisms observed in prion diseases. Further epidemiological evidence comes from the potential involvement of cerebral amyloid angiopathy (CAA), indicated by spontaneous intracerebral haemorrhage (ICH) between blood donors and recipients under prion-associated pathology (Zhao et al. 2023). Although these findings do not demonstrate direct transmissibility of prion proteins, they raise the possibility that amyloidogenic factors may circulate in the blood prior to clinical manifestation.

In parallel to the studies of human neuropathological conditions whose findings support the iatrogenic human-to-human transmission, more experimental studies have strengthened the interpretation by illustrating the capacity of amyloid-seeding in cell culture through humanised mice exposed to patient-derived brain material (Jaunmuktane et al. 2015). It is worth noting that complementary research on animal studies, including transmissible amyloidosis A outbreak in captive cheetahs revealed the presence of serum AA protein in faeces which was capable of inducing the same disease in mice. This points to a potential transmissible mechanism underlying the outbreak (Zhang et al. 2008). Collectively, the available evidence supports a hierarchical model of amyloid and prion transmission. At the highest level, rare iatrogenic exposures in humans—such as cadaver-derived growth hormone treatment and dural mater grafting—provide the strongest evidence for human-to-human transmission of seeding-competent pathology. Experimental studies further demonstrate that patient-derived material can transmit pathology from humans to animals under controlled conditions. At a broader level, natural outbreaks of AA amyloidosis in animals, such as those observed in captive cheetahs, indicate that amyloid diseases can propagate between individuals under specific environmental conditions. Together, these findings support a prion-like framework of amyloid propagation while suggesting that transmission occurs only under exceptional circumstances.

The identification of the [*PSI⁺*] prion’s self-propagating nature—where soluble Sup35 protein is converted into an aggregated, infectious conformation—was demonstrated through protein-only transmission experiments using recombinant Sup35 amyloid fibrils, which were shown to induce heritable prion states in yeast cells. Independent studies by King and colleagues and by Tanaka and Weissman provided direct evidence that purified Sup35 aggregates are sufficient to transmit prion phenotypes, establishing a mechanistic framework for protein-based inheritance (Tanaka et al. 2004, King and Diaz-Avalos 2004). These findings highlighted fundamental parallels between yeast and mammalian prion biology. Subsequent work further showed that the Sup35 prion domain fused to GFP (Sup35NM) can form transmissible aggregates in mammalian cells engineered to express the protein, indicating that the prion-forming region alone is sufficient to drive propagation across cellular contexts. Collectively, these studies demonstrate that prions can act as heritable protein-based elements, a concept that was first established in yeast, where the experimental tractability of the system enabled direct demonstration of protein-only inheritance.

## Yeast as a tool for research on, and treatment of, lysosomal storage disorders

### Lysosomal function and macromolecule degradation

Lysosomes are organelles enclosed by a phospholipid bilayer that contain an array of hydrolytic enzymes responsible for the degradation and recycling of a diverse range of biological macromolecules, including glycosaminoglycans, sphingolipids, oligosaccharides, nucleic acids, glycogen, complex lipids, and proteins (Kollmann et al. 2005, Parenti et al. 2015). Beyond their degradative role, lysosomes act as key regulators of cellular metabolism and signalling, maintaining homeostasis by recycling catabolic products—such as amino acids, nucleotides, sugars, and lipids—back into biosynthetic and energy-generating pathways. Cellular and foreign material destined for degradation reach lysosomes via endocytosis, phagocytosis, autophagy, or direct transport. The specific route depends on the origin and the nature of the cargo: extracellular substances are taken up through specialised endocytic mechanisms, including phagocytosis, micropinocytosis, clathrin-mediated endocytosis, clathrin-independent, and caveolin-dependent pathways. Meanwhile, intracellular components are primarily delivered via autophagy, a conserved process by which cytoplasmic material and damaged organelles are sequestered and transported to lysosomes for degradation and recycling (Lieberman et al. 2012).

### Lysosomal storage disorders and their molecular basis

Lysosomal storage diseases (LSDs) represent a group of more than 70 inherited metabolic disorders, most commonly resulting from autosomal recessive mutations, though some, such as mucopolysaccharidosis type II, Fabry disease, and Danon disease, follow an X-linked inheritance pattern (Meikle et al. 1999). They are characterised by lysosomal dysfunction that impairs the degradation of macromolecules. Disruption of these pathways results in the accumulation of storage material and the disturbance of signalling cascades, triggering cellular dysfunction, inflammation, and progressive pathology (Platt et al. 2018, Fiorenza et al. 2018). The clinical presentation varies widely—from severe congenital or infantile forms to milder, late-onset variants—with manifestations involving visceral, ocular, skeletal, haematological, and neurological systems. Notably, over 70% of LSDs exhibit neurological involvement, and those presenting in early development are typically the most severe. Representative examples include Gaucher, Tay–Sachs, and Niemann–Pick diseases (Beck 2001, Chin and Fuller 2022). LSD-associated neurological disorders are especially challenging to treat, as the therapeutic agents are required to overcome the blood-brain barrier to achieve significant therapeutic relief of the disease burden (Abbot and Williams 2022).

For this reason, a novel and more effective treatment plan for LSDs has never been more urgent. Given the complex nature of LSDs, particularly those with neurological conditions, a model system is especially required to understand the underlying mechanisms of LSDs. Similar to plant cells, budding yeast *S. cerevisiae*—also a eukaryote—contains a vacuole that mirrors many of the degradative and homeostatic functions of the lysosome in animal cells, enabling its use as a simplified model to study lysosomal storage disorders (Li and Kane 2009, Armstrong 2010).

### Enzyme replacement therapy and the central role of M6P

**Figure 2.**
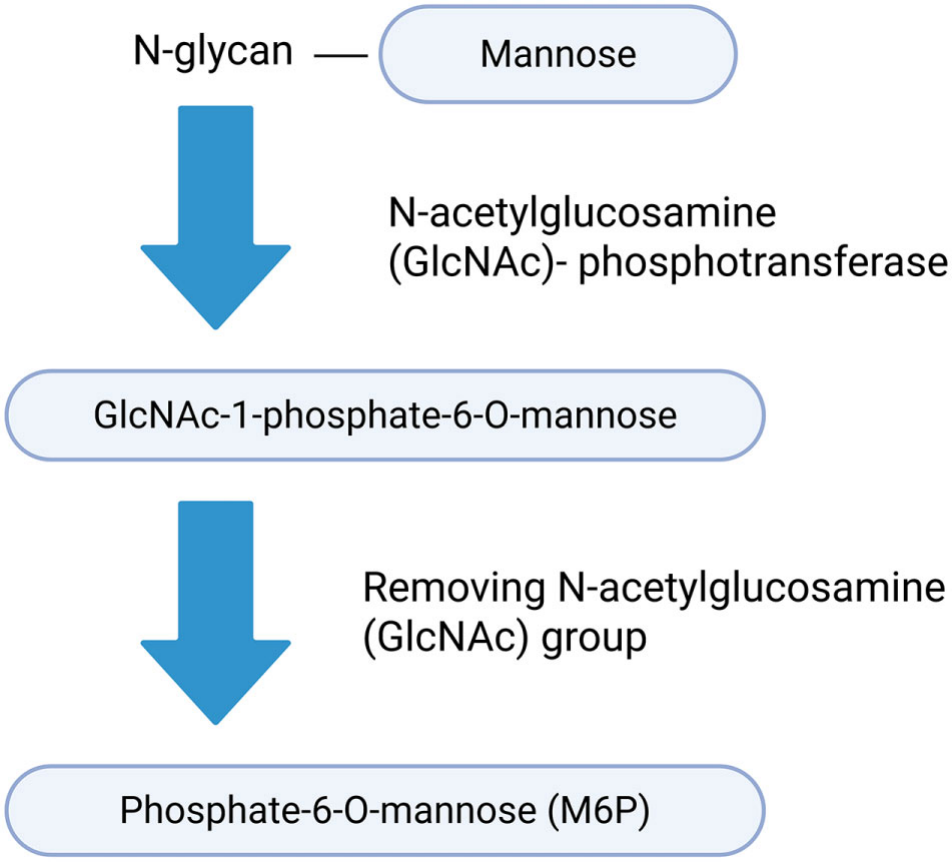
In mammalian cells, mannose-6-phosphate is produced to enable recognition by mannose-6-phosphate receptors located mainly in the trans-Golgi Network and endosomal compartments, which mediate the trafficking of lysosomal enzymes to lysosomes—a process that yeast naturally lacks. Created in BioRender. Do, A. (2026) https://BioRender.com/6xe8ohk

One of the strategies to treat LSDs is enzyme replacement therapy (ERT), in which deficiencies in lysosomal enzymes can be overcome by administering the enzyme to the diseased cell lysosomes (Desnick and Schuchman 2002). Lysosomal enzymes are glycoproteins typically synthesised in the endoplasmic reticulum and trafficked via the Golgi apparatus to lysosomes. With the exception of therapeutic enzymes for Gaucher disease, most others require N-glycans to bear mannose-6-phosphate (M6P), a modification that can be recognised by the mannose-6-phosphate receptors (MPRs) on the plasma membrane to enable receptor-mediated uptake and lysosomal delivery. As such, the M6P component of the therapeutic enzymes is a critical factor for efficient targeting and clearance of accumulated substrates in LSDs (Zhu et al. 2009, Tiels et al. 2012). In mammalian cells, N-glycans with the M6P modification are naturally generated through a two-step process: N-acetylglucosamine (GlcNAc)-phosphotransferase adds GlcNAc-1-phosphate to the 6-hydroxyl group of a specific mannose residue, forming GlcNAc-1-phosphate-6-O-mannose. This is then followed by the removal of the outer GlnNAc to leave a phosphate group linked to mannose (phosphate-6-O-mannose) or M6P (Fig. 2) (Coutinho et al. 2012, Probst Olivia et al. 2013).

### Engineering yeast to produce M6P-modified lysosomal enzymes

Yeast naturally lacks the M6P-containing glycan, required for efficient lysosomal targeting in ERT, however has other mannosylphosphate. To produce a human-compatible M6P modified protein, yeast can be engineered to carry mannosylphosphate groups, forming mannosylphosphorylated mannose (mannose-1-phosphate-6-O-mannose), which can be converted to M6P by enzymatically removing the outer mannose residue. This was accomplished through three important steps: (1) disruption of genes responsible for yeast-specific glycan synthesis, (2) overexpression of *MNN4* to enhance mannosylphosphorylation, and (3) in vitro removal of the outer mannose using bacterial enzymes to expose M6P (Chiba et al. 2002, Akeboshi et al. 2009). Noteworthy, a study from Tiels’ research group reported that glyco-engineered *Yarrowia lipolytica* produced α-glucosidase with over 15-fold higher M6P content than the currently approved Pompe disease therapy (alglucosidase alfa), resulting in more efficient lysosomal delivery and superior glycogen clearance in the heart and muscle of a Pompe disease mouse model (Tiels et al. 2012).

In *S. cerevisiae*, mannosylphosphate (not M6P) can be found at four positions within N-linked oligosaccharides—two located in the polymannose outer chain (at the non-reducing end and branch points) and two within the core oligosaccharide. In budding yeast, these modification is mediated by the coordinated activity of Mnn6p and Mnn4p (Jigami and Odani 1999). Mannosylphosphorylation of the outer mannose chain of N-glycans was catalyzed by Mnn6p protein, while Mnn4p protein function remained unclear. Consistent with this, overexpression of *MNN4* enhances mannosylphosphorylation independently of Mnn6p abundance, suggesting that Mnn4p acts as a rate-limiting factor. This conclusion is supported by the intensity of Alcian blue staining, a method used to detect negatively charged phosphomannan residues on the yeast cell wall. Yeast strains with higher levels of cell wall mannosylphosphate residues- such as wild-type *S. cerevisiae* or *MNN4*-overexpressing cells- exhibited stronger Alcian blue staining intensity, whereas *mnn4* mutants showed considerably reduced staining (Jigami and Odani 1999, Gil et al. 2015). These results not only clarify the genetic regulation of mannosylphosphorylation in *S. cerevisiae* but also provide a foundation for targeted glycoengineering strategies. Such approaches can be leveraged to generate human-compatible lysosomal enzymes in yeast, advancing both the study of lysosomal storage disorders and the development of improved enzyme replacement therapies.

## Yeast as a platform for study of mitochondria

### Mitochondrial structure and cellular functions

In eukaryotic cells, mitochondria are vital organelles that orchestrate cellular metabolic homeostasis, energy production, redox signalling, calcium buffering, and apoptosis (Song et al. 2024). Structurally, mitochondria possess a unique double-membrane architecture consisting of a relatively permeable outer membrane and a highly selective inner membrane that is extensively folded into cristae. These cristae increase the surface area available for oxidative phosphorylation and become sites of adenosine triphosphate (ATP) production (Kathiresan et al. 2025).

Beyond their structural organization, mitochondrial function is tightly regulated by dynamic processes, including fissions, fusions, biogenesis, and autophagy to preserve a balanced population of mitochondria in the cells (Osellame et al. 2012, Javadov et al. 2020). Mitochondria also play essential roles in various metabolic pathways, including the citric acid cycle, β-oxidation of fatty acids, and the production of vital metabolic precursors necessary for cellular growth (Spinelli and Haigis 2018).

### Mitochondrial dysfunction contributing to human genetic diseases

Since the pioneering work of Boris Ephrussi in 1949, yeast has emerged as a cornerstone model for investigating mitochondrial diseases. Its strong fermentative capacity allows survival despite mutations that impair oxidative phosphorylation or even the complete absence of mitochondrial DNA under anaerobic conditions, making it a robust framework for investigating mitochondrial dysfunction and its relevance to human disease. (Lasserre et al. 2015, Malina et al. 2018).

Mitochondria retain a small circular genome inherited from their bacterial ancestry (Ernster and Schatz 1981, Gray et al. 1999). Although most mitochondrial proteins are encoded in the nucleus, the mitochondrial genome is a starting point of multiple essential respiratory chain subunits as well as mitochondrial tRNA (Andersson et al. 1998). Early work by Gottfried Schatz and colleagues, largely carried out in yeast, established that these nuclear-encoded proteins are synthesised in the cytosol and subsequently imported into mitochondria, providing a mechanistic framework for mitochondrial biogenesis in eukaryotic cells (Schatz and Butow 1983). Therefore, mitochondrial biogenesis depends on the tight coordination between the nuclear and mitochondrial gene expression to ensure the correct assembly of the oxidative phosphorylation machinery (Anderson et al. 1981). When mitochondrial DNA (mtDNA) dysfunction occurs within the mitochondria, it is revealed to be connected to metabolic and cardiovascular disorders, brain injuries, inflammation, and age-related disorders due to its involvement in cell survival and death (Picard et al. 2016). Mutations in mitochondrial DNA are linked to various diseases, including Leigh syndrome, Pearson syndrome, Kearns–Sayre Syndrome, MELAS (Mitochondrial Encephalopathy, Lactic Acidosis, and Stroke-like episodes), and MERRF (Myoclonic Epilepsy and Ragged-Red Fibers) (Santorelli et al. 1993, Davidson and King 1997, Williams et al. 2012, Parikh 2016, Zeng et al. 2022). These mitochondrial diseases are serious conditions which have limited treatment options. Unlike nuclear genetic disorders, diseases caused by mtDNA are inherited through maternal pass down and do not follow a Mendelian inheritance pattern (Wei and Chinnery 2020). Their phenotypic expression is influenced by heteroplasmy—the coexistence of both wild-type and mutant mitochondrial within a cell. The percentage of mutant mtDNA is considered to be the disease severity determinant, where a higher proportion correlates with more pronounced clinical outcomes. Because mitochondrial disorders compromise oxidative phosphorylation, tissues that have high energy requirements and possess limited capacity to compensate through glycolysis are predominantly affected (Osellame et al. 2012). This suggested a strong dependence of these tissues on intact mitochondrial function.

### Yeast AAA-ATPases with homology to human paraplegin

Hereditary spastic paraplegia (HSP) is a genetically inherited neurodegenerative disorder that is characterised by a slow progressive degeneration of the distal axons, corticospinal and multiple nerve cells located in the spinal cord (Dürr et al. 1994). This disorder is identified to be associated with the *SPG7* gene product called paraplegin which comprises 795 amino acids. Paraplegin is a protein highly homologous to yeast ATP-dependent zinc metalloproteases, including Afg3p, Rca1p, and Yme1p (Pearce 1999). In *S. cerevisiae*, both Afg3p and Rca1p are necessary for respiratory growth and the proper formation of ATP synthase and respiratory chain complexes in the inner mitochondrial membrane (Tzagoloff et al. 1994, Paul and Tzagoloff 1995). These proteins belong to the ATPases Associated with diverse cellular Activities (AAA)-ATPase and use the energy derived from ATP hydrolysis to carry out critical mechanical functions within the cell, such as protein unfolding, degradation, membrane remodelling, and organelle maintenance. Importantly, human paraplegin performs a comparable role within the mitochondrial inner membrane, contributing to protein quality control and the maintenance of respiratory chain function.

Given this strong functional conservation, *S. cerevisiae* provides a powerful and genetically tractable system for investigating how mutations in *SPG7* disrupt mitochondrial proteostasis. In yeast, loss or dysfunction of Afg3p and Rca1p leads to defective assembly of respiratory chain complexes and impaired mitochondrial protein quality control, phenotypes that mirror the consequences of paraplegin deficiency in human cells (Pareek and Pallanck 2020). These defects result in compromised oxidative phosphorylation and reduced cellular energy production. Similarly, mutations in paraplegin are thought to impair the turnover and maturation of mitochondrial proteins, leading to the accumulation of misfolded or dysfunctional proteins within the inner mitochondrial membrane (Wedding et al. 2014).

Such mitochondrial dysfunction is particularly detrimental to neurons, which rely heavily on efficient energy production and are highly sensitive to disruptions in proteostasis. Consequently, impaired paraplegin function contributes to the progressive neurodegeneration observed in HSP.

## Use of yeast in drug discovery

Yeast, especially *S. cerevisiae*, is an invaluable tool for the discovery and development of therapeutic agents as it allows insights into the efficacy, potency and mode of action of compounds. In a study, Tebbets and colleagues developed a high-throughput bioassay to screen microbial extracts for novel antifungal compounds. Using this approach, they identified a class of natural products known as macrotetrolides, which exhibited promising antifungal activity (Tebbets et al. 2013). Another example is the discovery of multiple natural product compounds through screening various marine species for the ability of their extracts to cure yeast prions. The study by Jennings et al. discovered several valuable compounds with different efficacy and toxicity profiles. This study found that the Verongidae sponges potentially harbour members of a compound class that, apart from the ability to cure yeast prions, also possesses a wide range of activities, including anti-microbial, anti-HIV, acetylcholinesterase inhibition, histamine H_3_ receptor inhibition, and apolipoprotein E modulating activity (Jennings et al. 2018). These findings illustrate how yeast-based prion screening can uncover bioactive compounds from complex natural product mixtures, highlighting the value of yeast as a system for functional discovery. Such approaches would be considerably more challenging to implement in more complex biological systems.

### Expanding the role of yeast in drug discovery through genome-wide gene knockout screening

To further expand the utility of yeast in drug discovery, yeast gene knockout collections have been widely employed in chemical genomics to elucidate the mechanism of action of small molecules. In fact, the completion of the *S. cerevisiae* genome was achieved in 1995, marking the first fully decoded eukaryotic genome. This later paved the way for the development of a comprehensive yeast gene knockout collection in 2002. The gene knockout collection has since served as an essential resource for probing drug mechanism, and target identification (Hoon et al. 2008, Giaever and Nislow 2014). The yeast deletion collection or yeast knockout set (YKO) consists of more than 21 000 *S. cerevisiae* mutant strains that carry precise start-to-stop deletions of ∼6000 open reading frames, covering nearly all non-essential genes. The collection includes heterozygous and homozygous diploids, as well as haploid strains of both *MAT***a** and *MAT*α mating types (Giaever et al. 1999). The effectiveness of the YKO has been exemplified by a report by Parsons and colleagues, where they successfully established a chemical-genetic interaction profile for 82 pure compounds and natural product extracts which highlighted compound mechanism-of-action as well as their cellular pathways and proteins affected by the compounds or extract. This approach identified two antifungal natural product extracts, one from a sea cucumber and the other from a marine sponge, that produced similar fitness profiles, suggesting they might have had a shared mechanism of action despite their distinct biological origins. The strong overlap in profiles between two marine extracts indicates the potential of yeast knockout screening to uncover conserved bioactivities hidden in compounds from taxonomically distinct sources (Parsons et al. 2006).

### Use of the yeast two-hybrid screen for protein-protein interaction mapping

Beyond drug discovery, understanding disease-related cellular processes at the molecular level requires detailed knowledge of protein–protein interactions. Mapping these interactions reveals potential drug targets, paving the way for the development of small molecules or other biologics that can modulate them (Moosavi et al. 2017). An illustration of this is the study by Jia et al., where they used the yeast two-hybrid technique to profile snake venom protein interactions. Snakebite has been recognised as a significant public health concern, as snake venom is a cocktail of distinct proteins and peptides that interact synergistically with each other to elicit toxic responses. By applying the yeast two-hybrid analysis, this study examined interactions between venom components themselves, proposing that phospholipase A_2_s (PLA_2_s)—one of the most common snake venom enzymes—are capable of self-interacting, and that PLA_2_ lysine-49 interacts with venom cysteine-rich secretory protein (CRISP) (Jia et al. 2021). These intrinsic interactions contribute to the stabilization of toxin complexes and modulation of their biochemical activity, thereby enhancing the potency of the venom. More broadly, this study illustrates how yeast-based interaction mapping can uncover mechanistic relationships between proteins, providing insights that may guide the development of more targeted and effective therapeutic interventions, such as improved antivenom strategies.

## Challenges and limitations

*Saccharomyces cerevisiae* has been extensively utilised as a model organism to elucidate cellular mechanisms and disease pathways through advanced technologies such as gene deletion libraries, heterologous expression systems, and high-throughput therapeutic screening. Nevertheless, even with these advances, yeast-based research still faces several inherent limitations when translating findings to human systems (Mohammadi et al. 2015, Cervelli and Galli 2021).

Notably, the direct translation between yeast and human protein function has proven to be a challenging task. In particular, mitochondrial functions have been demonstrated to be more complex in humans, compared to yeast (Lasserre et al. 2015, Malina et al. 2018). While yeast mitochondria share conserved respiratory and proteostatic machinery with humans, yeast cells can survive severe mitochondrial dysfunction due to their strong fermentative capacity and tolerance of mitochondrial DNA loss. In *S. cerevisiae*, energy production can be maintained through fermentative glycolysis, allowing cells to generate ATP independently of oxidative phosphorylation. As a result, yeast can survive the complete loss of mitochondrial DNA or defects in the electron transport chain without immediate lethality (Lasserre et al. 2015). In addition, mitochondrial dysfunction in yeast primarily affects respiratory growth rather than viability, enabling the study of otherwise deleterious mutations. In contrast, mitochondrial defects in humans disrupt energy production, apoptosis, and metabolic signalling in a tissue-dependent manner, often resulting in severe or lethal disease. Moreover, yeast studies are typically conducted in genetically uniform (isogenic) strains, whereas human populations exhibit substantial genetic heterogeneity, which can influence disease severity and phenotypic variability. Thus, phenotypes observed in yeast may underestimate the physiological impact of mitochondrial mutations in human systems. Therefore, some defects observed in yeast mitochondria could potentially have broader and more unpredictable consequences in human mitochondrial systems.

Although yeast provides powerful insights into conserved cellular mechanisms, differences between yeast and human biology impose disease-specific constraints on modelling and interpretation. In the context of prion diseases, prion propagation is not restricted to a single system, as prion proteins can maintain their aggregated, self-templating conformations across species boundaries. Yeast prion domains have been shown to remain stable in their prion form when expressed in mammalian cells, while mammalian prion proteins can likewise retain prion-like properties in yeast, indicating that key aspects of the propagation machinery are conserved (Johnson et al. 2008, Chernoff et al. 2020). Consistent with this, several compounds identified as curing yeast prions have also demonstrated activity in mammalian systems, and vice versa, suggesting a degree of conservation in the underlying molecular targets. However, yeast prions are typically non-lethal and lack the neuronal specificity, neuroinflammatory responses, and progressive tissue degeneration that define mammalian prion disorders. A further limitation of yeast prion models lies in the temporal dynamics of disease. Yeast prions propagate rapidly and produce readily detectable phenotypes, making them highly tractable for laboratory study. In contrast, mammalian prion diseases are characterised by prolonged incubation periods that can span years or decades, during which pathogenic protein conformers accumulate silently before overt disease manifests. For this reason, yeast models are unable to recapitulate the extended preclinical phase of prion diseases, where prions may be present without causing overt pathology. As a result, they cannot readily distinguish between asymptomatic prion presence and pathogenic progression, limiting their ability to model disease latency and long-term neurodegenerative outcomes. Consequently, yeast systems are best suited for dissecting the molecular mechanisms of prion propagation rather than modelling disease toxicity or clinical progression.

For lysosomal storage disorders, the yeast vacuole serves as a functional analogue of the mammalian lysosome and allows precise analysis of intracellular trafficking, autophagy, and macromolecular degradation. However, yeast lacks the multicellular architecture required to model tissue-specific storage pathology, immune activation, and neurological involvement that characterise many LSDs. As a result, yeast systems capture cell-autonomous defects but cannot reproduce multicellular processes like immunity and neuronal communication, or organ-level disease manifestations (Bilinski et al. 2017).

Another major limitation of using yeast as a model system lies in its differences in protein processing and post-translational modifications compared to human cells. While both perform N-linked glycosylation, yeast predominantly generates highly mannosylated N-glycans, which differ from the complex-type glycans found in humans that contain terminal galactose and sialic acid residues. These differences can influence glycoprotein folding, stability, and biological activity, thereby complicating studies of human proteins that depend on precise glycosylation for proper function (Trimble et al. 2003, Moremen et al. 2012, Hamilton and Zha 2015). Similarly, yeast metabolises drugs through pathways that often yield metabolites distinct from those in human systems, posing challenges for assessing pharmacokinetics, toxicity and therapeutic index. Advances in synthetic biology and glycoengineering—particularly the development of *Pichia pastoris* strains capable of producing human-like glycan structures—are allowing yeast to perform human-like glycosylation, enabling the production of recombinant proteins and therapeutic enzymes with more clinically relevant post-translational modifications (Karbalaei et al. 2020). While such engineering significantly improves the utility of yeast in biopharmaceutical applications, fundamental metabolic differences remain an important limitation for modelling drug metabolism and safety in humans (Kokina et al. 2019).

## Future perspectives and conclusions

Despite these limitations, yeast remains an invaluable model for study of fundamental cellular processes, protein-protein interactions and for use in drug discovery. What is now needed is more advanced approaches in genetic engineering and synthetic biology that may potentially overcome many of the current challenges associated with using yeast in biomedical research. The application of humanised yeast strains, where certain human genes and pathways are introduced into yeast, is a promising strategy to enhance the relevance of the yeast model in research on disease mechanisms and drug-target interactions.

A promising direction for future research involves integrating yeast-based systems with complementary mammalian assays to better bridge the gap between fundamental discoveries and clinical applications. While yeast offers a powerful and tractable platform for studying cellular processes, its limitations—particularly in replicating human-specific biology—necessitate validation through additional models. Advances such as the development of humanised yeast strains, improved modelling of post-translational modifications, and integration with complementary mammalian systems are expected to enhance the physiological relevance of yeast studies and improve their translational value. Such integrative approaches not only expand the utility of yeast in biomedical research but also contribute to a more comprehensive understanding of human diseases and the development of novel therapeutics.
